# Septic Arthritis and Osteomyelitis in Finger Caused by *Mycoplasma phocimorsus* from Brown Bear, Alaska, USA

**DOI:** 10.3201/eid3107.250419

**Published:** 2025-07

**Authors:** Benjamin P. Westley, Alan Swenson, Shanna Berry-Vo, Stephanie Wettin, Daniel R. Brown

**Affiliations:** Private practice, Anchorage, Alaska, USA (B.P. Westley, S. Berry-Vo); Orthopedics Physicians of Alaska, Anchorage (A. Swenson, S. Wettin); College of Veterinary Medicine, University of Florida, Gainesville, Florida, USA (D.R. Brown)

**Keywords:** Mycoplasma, zoonoses, bacteria, osteomyelitis, finger, seal, bear, Alaska, United States

## Abstract

*Mycoplasma phocimorsus* is an identified zoonotic agent of musculoskeletal infections. Osteomyelitis developed in a patient after injury sustained while skinning a bear, and he experienced delayed diagnosis after ineffective treatments. Clinicians should use doxycycline or moxifloxacin therapy in treatment-refractory cases with exposure to seals, cats, or bears while awaiting molecular diagnostics results.

In September 2024, a 29-year-old hunter sought care 7 days after he lacerated his left fifth finger while skinning a brown bear (*Ursus arctos*) on the Alaska Peninsula near Ivanof Bay, Alaska, USA. The injury occurred when separating paw bones to free skin. His hand and knife contacted the bear’s mouth before injury, but the intestinal tract was not penetrated. He reported 3 days of redness and painful swelling over the proximal interphalangeal (PIP) joint. He was placed on oral trimethoprim/sulfamethoxazole (160 mg/800 mg) and topical mupirocin (2% cream, 3×/d). On day 5 of illness, he developed fever and tachycardia and was admitted to a hospital. His temperature was 38.2°C and pulse 108 beats/min; blood pressure and respiratory rate were unremarkable. A laceration with scant drainage but substantial edema was present over the dorsal left fifth PIP joint (Figure 1). Peripheral leukocyte count was 8,300 cells/μL (reference range 4,500–11,0000 cells/μL), and C-reactive protein level was 11.6 mg/L (reference range 0.2–3.0 mg/L). Radiographs showed soft tissue swelling without bone or joint abnormality. 

**Figure 1:**
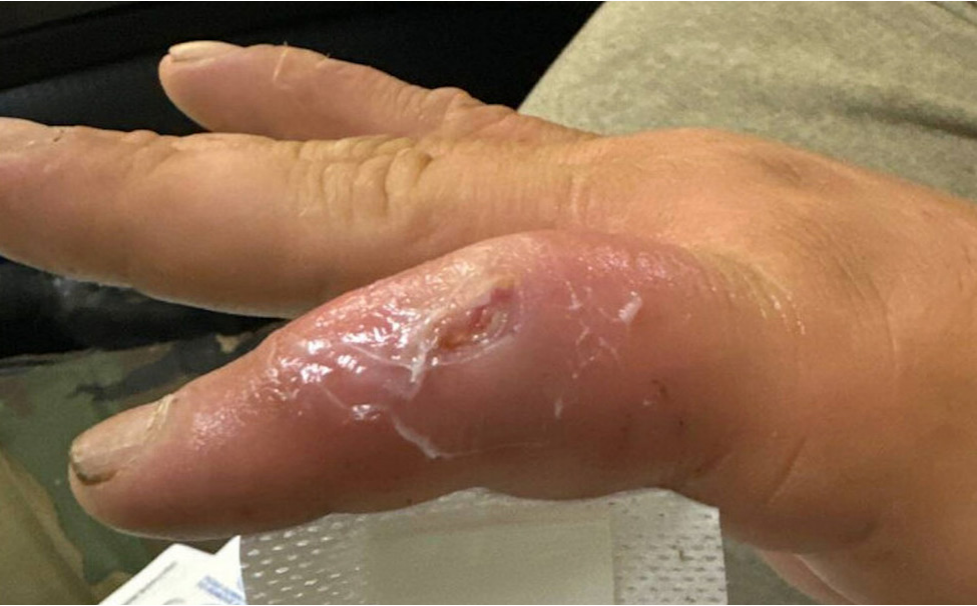
Finger laceration in case of septic arthritis and osteomyelitis in finger caused by *Mycoplasma phocimorsus* from brown bear (*Ursus arctos*), Alaska, 2024. Left fifth finger laceration occurred while skinning a brown bear. Laceration is shown overlying the proximal interphalyngeal joint with surrounding edema.

We administered 1 dose of ceftriaxone (2 g), followed by piperacillin/tazobactam (3 g/375 mg every 8 h) and vancomycin (1.25 g every 8 h) for 3 days. We performed surgery on day 6 of illness and noted left fifth finger PIP septic arthritis and necrotic extensor tendon disruption. Gram stains of debrided tissue revealed no organisms. We inoculated tissue on tryptic soy agar with 5% sheep blood, chocolate blood agar, and MacConkey agar plates and in chopped meat broth and prepared *Brucella* blood agar, phenylethyl alcohol blood, and *Bacteroides* bile esculin and laked *Brucella* blood agar with kanamycin and vancomycin plates. We saw no growth after 7-day incubation. Blood monitored for 5 days on a BACTEC-FX blood culture system (Becton Dickinson, https://www.bd.com) grew no organisms. Fever resolved on day 6 of illness, and on day 8 of illness, we discharged the patient with trimethoprim/sulfamethoxazole (160 mg/800 mg 2×/d for 21 d) and amoxicillin/clavulanate (875 mg/125 mg 2×/d for 21 d).

On day 46 of illness, the patient had repeat debridement of the finger because of persistent swelling and magnetic resonance imaging evidence of PIP joint osteomyelitis (Figure 2). Leukocyte count was 10,300 cells/μL, erythrocyte sedimentation rate was 5 mm/h (reference range <15 mm/h), and C-reactive protein level was 8.6 mg/L. Vancomycin and piperacillin/tazobactam treatment was repeated concurrently for 3 days. At surgery, we found substantial cartilage and bone erosive changes in the PIP joint. Gram stain of operative tissue revealed no organisms, and we detected no growth in conventional aerobic and anaerobic bacterial cultures after 7-day incubation. Fungal cultures inoculated on Sabouraud and brain-heart infusion agar showed no growth. On day 49 of illness, doxycycline (100 mg 2×/d) was started. 

**Figure 2:**
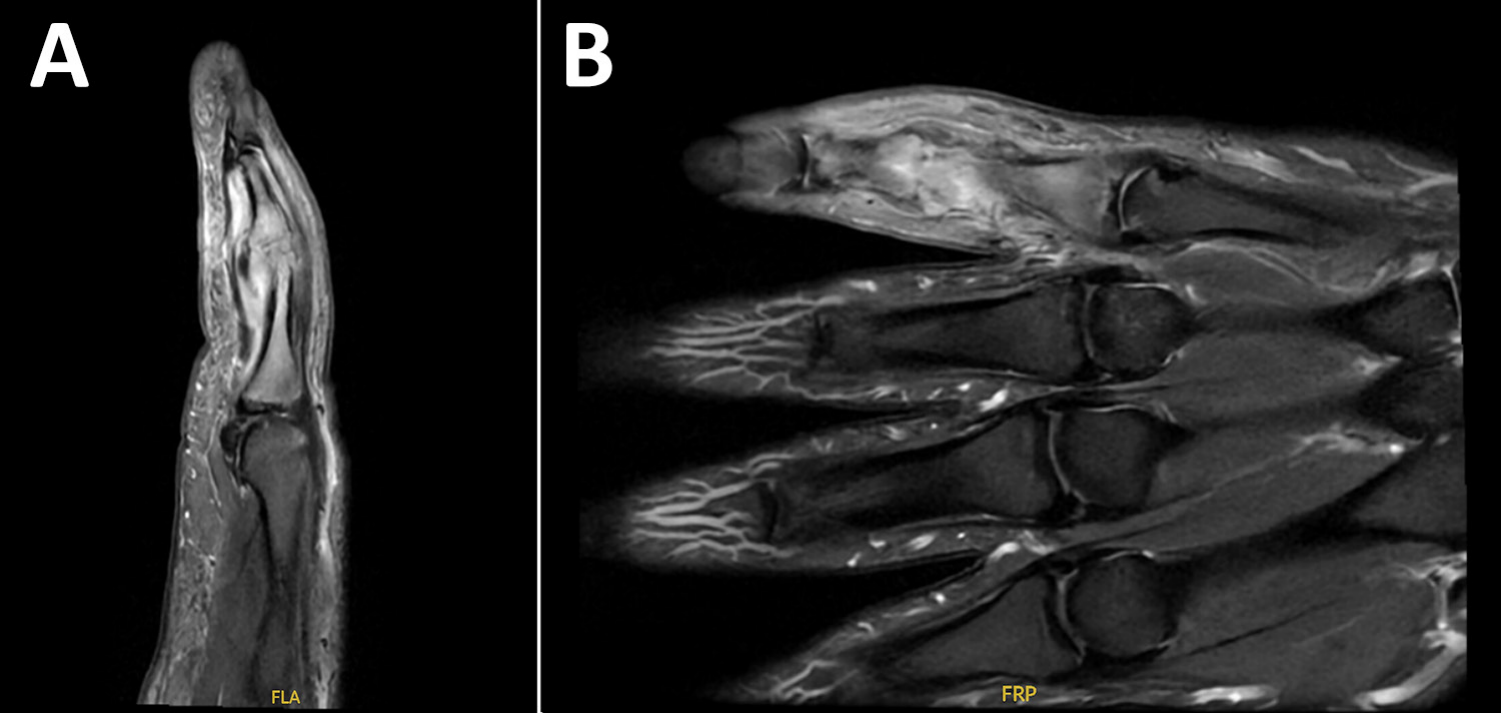
Magnetic resonance imaging in case of septic arthritis and osteomyelitis in finger caused by *Mycoplasma phocimorsus* from brown bear (*Ursus arctos*), Alaska, 2024. Left fifth finger sagittal (A) and coronal (B) images demonstrate edema of the proximal and middle phalanges on short tau inversion recovery sequences, suggestive of osteomyelitis.

On day 53 of illness, tissue from surgery sent to the University of Washington Molecular Microbiology Laboratory for broad-range 16S rDNA PCR bacterial detection revealed *Mycoplasma phocimorsus* (GenBank accession no. PV641041) using methods described elsewhere (*1*). The amplified 315-nt sequence was 100% identical with the 16S rRNA gene sequence of *M. phocimorsus* type strain M5725. 

In December 2024, the patient completed 42 days of doxycycline according to standard treatment guidelines for osteomyelitis. Although pain and swelling improved, decreased range of motion persisted at the end of therapy. One month later, he had no signs or symptoms of relapsing infection.

Seal finger was described in 1907 as a painful swollen finger that developed after an injury when butchering seals (*2*). In 2014, several authors treated a ringed seal (*Pusa hispida*) hunter in Alaska for seal finger and hip septic arthritis caused by a *Mycoplasma* species detected by 16s rRNA sequencing, but they could not propogate the organism in culture (*3*). Nine years later, researchers isolated 6 independent strains of a *Mycoplasma* species from patients from Scandinavia after contact with seals and proposed the name *M. phocimorsus* (*4*). Strains were susceptible to doxycycline and moxifloxacin and showed >99.5% rRNA similarity with the sequence identified in the seal hunter from Alaska, confirming *M. phocimorsus* is present in Alaska. Subsequently, *M. phocimorsus* was identified as the cause of tenosynovitis after a cat scratch (*5*). Our patient had no exposure to seals and minimal exposure to a dog and cat owned by his mother.

Harbor seals (*Phoca vitulina*), sea otters (*Enhydra lutris*), and Steller sea lions (*Eumetopias jubatus*) are encountered in that region of Alaska (D.D.W. Hauser, University of Alaska, pers. comm., email, 2024 Mar 14). Brown bears are voracious hunters and scavengers, so this case may reflect transient colonization after predation of an infected seal or other animal. However, *Mycoplasma* species occur in brown bear gut microbiomes (*6*), suggesting that brown bears may be frequent reservoirs of *M. phocimorsus.* As in previous cases, this patient experienced delayed diagnosis and ineffective treatments because of *Mycoplasma* species’ resistance to most antibiotic drugs and inability to be grown in conventional cultures. Clinicians should remain alert to the possibility of *M. phocimorsus* infection after exposure to seals, cats, or bears and initiate doxycycline or moxifloxacin therapy while awaiting confirmatory molecular testing, particularly in treatment-refractory infections.
